# Multidimensional voiceprint feature assessment system for identifying the depression in children and adolescents: a diagnostic test

**DOI:** 10.3389/fpsyt.2023.1105534

**Published:** 2023-05-10

**Authors:** Jie Luo, Mengqi Liu, Lihui Feng, Zhaojun Li, Yuanzhen Wu, Jihua Lu, Fan He

**Affiliations:** ^1^National Clinical Research Center for Mental Disorders, Beijing Key Laboratory of Mental Disorders, Beijing Anding Hospital, Beijing Institute for Brain Disorders, Capital Medical University, Beijing, China; ^2^Beijing Institute of Technology, School of Optics and Photonics, Beijing, China; ^3^Beijing Institute of Technology, School of Integrated Circuits and Electronics, Beijing, China

**Keywords:** major depressive disorder, children and adolescents, multidimensional voiceprint feature assessment system, objective diagnosis, diagnostic accuracy

## Abstract

**Objective:**

We designed a diagnostic test to evaluate the effectiveness and accuracy of a multidimensional voiceprint feature diagnostic assessment (MVFDA) system vs. the 24-item Hamilton Rating Scale for Depression (HAMD-24) for adjunctive diagnosis of children and adolescents with major depressive disorder (MDD).

**Methods:**

This study included 55 children aged 6–16 years who were clinically diagnosed with MDD according to the DSM-5 and analyzed by professional physicians, and 55 healthy children (typically developing). Each subject completed a voice recording and was scored on the HAMD-24 scale by a trained rater. We calculated the validity indices, including sensitivity, specificity, Youden's index, likelihood ratio, and other indices including predictive value, diagnostic odds ratio, diagnostic accuracy, and area under the curve (AUC), to assess the effectiveness of the MVFDA system in addition to the HAMD-24.

**Results:**

The sensitivity (92.73 vs. 76.36%) and the specificity (90.91 vs. 85.45%) of the MVFDA system are significantly higher than those of the HAMD-24. The AUC of the MVFDA system is also higher than that of the HAMD-24. There is a statistically significant difference between the groups (*p* < 0.05), and both of them have high diagnostic accuracy. In addition, the diagnostic efficacy of the MVFDA system is higher than that of HAMD-24 in terms of the Youden index, diagnostic accuracy, likelihood ratio, diagnostic odds ratio, and predictive value.

**Conclusion:**

The MVFDA has performed well in clinical diagnostic trials for the identification of MDD in children and adolescents by capturing objective sound features. Compared with the scale assessment method, the MVFDA system could be further promoted in clinical practice due to its advantages of simple operation, objective rating, and high diagnostic efficiency.

## Introduction

Major depressive disorder (MDD) is a type of common mental illness. MDD could become the first-class diseases for the heavy burden worldwide by 2030 (1). However, the prevalence of depression tends to increase among adolescents. In the United States, the lifetime prevalence of MDD among adolescents aged 13 to 18 years is 11.0%, and the 12-month prevalence probability is 7.5% (2). In addition to the high probability of prevalence, the cure rate for adolescent depression is extremely low, and the suicide rate is quite high (3, 4). Moreover, as children and adolescents are in a crucial period of psychophysiological development, their depression has a more serious impact on the performance of both academic and social functions (5, 6). Therefore, early diagnosis and identification of MDD are essential.

There are two major diagnostic systems for depression: the DSM-5 (the Diagnostic and Statistical Manual of Mental Disorders) and the ICD (the International Classification of Diseases) systems. The recognition of depression depends on some basic clinical symptoms. In addition, clinicians will also assist in the diagnosis according to some classical scales, such as the HAMD (7), which can assess the severity of depression. The establishment of the HAMD depression scale includes some basic symptoms of depression, such as decreased interest and fatigue, and some additional symptoms like anxiety/somatization, cognition, and round-the-clock changes (7). However, these scales require rich clinical experiences (8, 9). Even senior physicians are prone to misdiagnosis when patients atypically conceal their illness or symptoms. Moreover, compared with adults, the clinical manifestations of children and adolescents with depressive disorders are more atypical, and the degree of coordination is lower, which makes it more difficult for clinicians, especially primary care medical workers, to identify adolescents with the depressive disorder (10–12). Considering the shortcomings of the scales, research on the objective adjunctive diagnosis of MDD patients has become a hot topic.

Patients with depression display different physiological indicators from healthy individuals, such as altered body posture, facial expressions, and voice. Researchers have extracted some specific features to diagnose depression. Studies show that the voice of patients with depression can change significantly; for instance, voice speed is extremely slow, and pauses are longer, more rigid, and more frequent (13–15). With the development of artificial intelligence (AI), it is easier to design Al-related algorithms based on the voice features of patients with depression that could help identify some classical symptoms of MDD (16). In addition, compared to other traits, sound acquisition is easier and cheaper (15). Therefore, researchers have shown great interest in speech recognition research (SDR) on depression. The current research on depression recognition employing speech includes two main aspects: (1) Analyzing the speech features of patients with depression. (2) Building a speech depression recognition model. Early studies mainly focused on the classical and related features of depression patients. Most studies analyzed the features of their speech in the time domain. Szabadi et al. found that the pause in the voice of depressed patients was prolonged, but the phonation period remained constant (17). Moreover, the length of their voice pause decreased with the improvement of clinical symptoms. Then, Greden and Carroll also confirmed Szabadi's conclusions (18). Hollien showed that patients with depression speak slower than healthy individuals, with a more monotonous intonation (19). However, the features extracted from the time domain are greatly affected by individual differences and cannot fully represent the features of patients with depression.

In addition, researchers have committed to investigating the changes in various acoustic features of speech signals, such as prosody, sound source, composition, and spectrum. The differences between the two types of people in the features of speech signals, such as fundamental frequency (F0), Mel cepstrum coefficient (MFCC), energy, frequency, and formant, have also been studied. Based on these features, a recognition model of depression has been constructed. In recent years, great advancements in SDR have been made possible thanks to the integration of acoustic features. Ooi et al. distinguished depressed adolescents from healthy individuals with a classification accuracy of 73% using four features (progressive, gross, TEO, and spectrum). The recognition effects of multi-channel classification with a weighted decision procedure are superior to all classifications based on a single feature or single channel (20). Cummins et al. combined features of MFCC and formant and used the GMM classifier to obtain an accuracy of 79% (21). Mendirata et al. obtained an accuracy of 80.67% using MFCC features and conducted principal component analysis (PCA), and clustering classification (22). These studies show that the designed recognition system based on the vocal differences of patients with depression has strong feasibility. For the experimental paradigms, the size of the data set and other aspects are difficult to compare horizontally with the results. Therefore, a general and optimal model with higher accuracy is very important. However, the conclusions drawn from studies on the same sound features are not consistent. For example, the experimental results of some studies show that the size of F0 is related to the severity of depression, while other experiments reveal that there is no correlation between F0 and depression (23). Even for the same group of subjects, the relationship between F0 and depression is also affected by the speech content. Therefore, finding a more accurate and scientific diagnostic system is very important. All told, the existing data sets are mostly focused on adults, and the studies are also inconsistent. There are fewer studies on the identification of adolescent speech than there are on adult speech. Due to the physical development of adolescents, the sound of children and adolescents is somewhat different from that of adults in terms of the frequency range, pitch, speech rate, fundamental frequency, resonance peak, and so on. For example, the vocal cords and throat structure of teenagers are not yet fully mature, so their voices tend to have a higher frequency range. In contrast, the voices of adults are more stable, with a relatively smaller frequency range. Teenagers' pitch is usually higher and sometimes less stable, while adults tend to have a more even and stable pitch. In addition, teenagers generally speak at a faster pace than adults, which may be related to their more active bodies and faster thinking ability. Therefore, to obtain a higher identification accuracy, it is essential to specifically recognize adolescent speech.

In response to the above problems, Beijing Anding Hospital and Beijing Institute of Technology have developed a new multidimensional voice feature diagnostic assessment (MVFDA) system that overcomes the shortcomings of the state-of-the-art algorithms and greatly improves the recognition accuracy based on features extracted from voiceprint. Furthermore, we specifically evaluated the effectiveness of this diagnostic system and compared it with the HAMD clinical scales.

## Methods and materials

### Study design

Aiming at diagnosing and evaluating the effectiveness of the MVFDA system for MDD and comparing it with the HAMD scale, the design of the trial follows the STARD statement. PASS15 software (NCSS, Kaysville, Utah, USA) was used to calculate samples. Referring to previous related studies, it has been shown that machine learning models have promising results with AUC values generally greater than 0.9. Under the conditions of α = 0.05 (unilateral), β = 0.1, and a 1:1 ratio between groups, 55 subjects were enrolled in each group of the MDD and healthy control (HC) groups. Each subject was evaluated and compared using the MVFDA system and HAMD scale.

### Subjects

Subjects in the MDD group were inpatients at the Beijing Anding Hospital. The inclusion criteria were as follows: (a) meet the DSM-5 diagnostic criteria for MDD; (b) be between the ages of 6 and 16, with no gender restriction; (c) be able to cooperate to complete the study; and (e) sign the informed consent. Exclusion criteria were as follows: (a) severe physical illness, such as pharyngeal edema, pharyngeal foreign bodies, hoarseness; (b) comorbid other psychiatric disorders, such as bipolar disorder and schizophrenia, and developmental disorders (e.g., autism spectrum disorder, intellectual impairment); and (c) other conditions deemed inappropriate for inclusion in the group by the investigators. Healthy controls were recruited from the community. Typically developing children and adolescents aged 10 to 18 years with no other conditions, and who were able to cooperate to complete all the requirements were recruited from the community and schools. Ultimately, we successfully recruited 110 subjects according to the study plan, with no subjects dropping out midway through. The project was conducted in accordance with the ethical standards of the Declaration of Helsinki and its subsequent amendments and was approved by the Ethics Committee of the Beijing Institute of Technology (Ethical number: BIT-EC-H-2022120). All subjects and their family members signed an informed consent form prior to the trial. All subjects were able to comply with the MVFDA system and HAMD assessment requirements during the study, and the data collected were valid and reliable.

### Gold standard

MDD is diagnosed according to the recommended guidelines and is based on the patient's medical history, clinical symptoms, disease course, and relevant examinations. In this study, two senior experts conducted detailed clinical interviews with each subject, obtained their medical history, performed a psychiatric evaluation, and combined these to form a diagnostic opinion according to the DSM-5 diagnostic criteria. Finally, the unanimous opinion of the two experts served as the gold standard for the complete diagnosis.

### MVFDA system

This algorithm achieves the recognition of depression based on voice data. Specifically, as shown in Table 1. First, multi-dimensional features are extracted from voice data, including energy-related, spectral, voice-related, and statistical information. Low-level descriptors (LLDs) of voiceprints are manually designed and generally calculated from a frame of voice. Various statistical functions are then calculated based on the LLD. Then, a more comprehensive feature set is constructed, and features with significant differences between classes are retained through feature screening. The next step is to extract features from the transform domain to optimize their differences. Finally, the integrated classification method is used to achieve high classification accuracy.

**Table 1:** Description of the features applied in the MVFDA system.

**Types of features**		**Names of features**	**Meaning**
LLD	Energy-related LLD	Sum of the auditory spectrum	The auditory spectrum includes information from the time and frequency domains of a sound signal
		RMS energy	Root mean square value of all samples in a frame
		Zero crossing rate	Number of times the signal crosses the horizontal axis
		Sum of RASTA-style filtered auditory spectrum	Auditory spectrum after RASTA filtering
	Spectral LLD	Mel Frequency Cepstrum Coefficient (MFCC) Energy, variance, and kurtosis of the spectral	Coefficients of Mel frequency cepstrum Energy and variance of spectral, and kurtosis of each spectral line
	Voice-related LLD	Fundamental frequency (F0) Jitter and shimmer Harmonics-to-Noise ratio (HNR)	The frequency of the fundamental tone in a polyphony Basic frequency and amplitude change of acoustic wave between adjacent periods Ratio of harmonic to noise components
Statistical features		Mean, Maximum, Minimum Variance Linear regression slope	Average, Maximum, Minimum, and Variance of samples Slope of linear regression line

### HAMD scale

The most widely used scale for assessing depression was created in 1960. There are three versions of this scale: the 17-item, 21-item, and 24-item. In this study, we used the 24-item HAMD. The HAMD scale is administered and independently scored by two trained raters. Most of the items are rated on a 5-point scale (0 to 4), and a few items are rated on a 3-point scale (0 to 2). Items 8, 9, and 11 of the scale are rated based on observation of the patient; the remaining items are rated based on the patient's own verbal narrative; item 1 requires a combination of the two. In addition, for items 7 and 22, information has to be collected from the patient's family or ward staff, while item 16 is based on weight records and can also be rated on the basis of the patient's complaints and information provided by their family or ward staff. According to Davis JM's delineation score of 24 items total, major depressive disorder is possible with a score >20. The scale is widely used in clinical practice and has high reliability and validity (24–27). All interviewers had passed the HAMD-24 consistency training. Additionally, the group's intraclass correlation coefficient (ICC) was higher than 0.8.

### Processing

We used the recording function of the MacBook Air 2020 to gather the voice data of all subjects. The data were collected in a quiet room at Beijing Anding Hospital to evaluate the MVFDA system. First, the entire evaluation process was briefly introduced and described. Then, the investigator read part of the story or some words to demonstrate. The recording was initiated once the participant comprehended the lists that needed to be read. Children were required to read one story and six groups of words in succession. For the purpose of feature extraction and classification, speech differences between depressed adolescents and healthy ones were applied. A 10-min break was taken after completing the MVFDA system assessment, and then two professional raters assessed them using the HAMD scale.

### Statistical analysis

Data were analyzed using SPSS25.0 software (IBM, Armonk, NY, USA). Age differences between the two groups were analyzed by *t*-test, and gender differences were analyzed by chi-squared. Descriptive statistics were conducted to analyze the medication use of patients in the MDD group. The consistency of the results between the two HAMD scale raters (ML and JLuo) was determined by kappa analysis. A series of indicators, including sensitivity, specificity, the Youden index, diagnostic odds ratios, likelihood ratios, predictive values, and diagnostic accuracy, were calculated to evaluate the diagnostic validity of MVFDA compared with HAMD. Drawing the ROC curve, calculating the area under the curve (AUC), and applying Z-tests should all be performed to test for differences in the AUC, as it is generally believed that the closer the AUC is to 1, the more reliable the test method is.

## Results

### Subject characteristics

A total of 55 subjects, 20 boys and 35 girls, with a mean age of 14.40 ± 1.72 years, were included in the MDD group of this trial. A total of 55 subjects, 22 boys and 33 girls, with a mean age of 14.83 ± 1.50 years, were included in the HC group. There was no statistical difference in age (*p* = 0.16) or gender (*p* = 0.43) between the two groups. The medication use of the patients in the MDD group is shown in Table 2.

**Table 2:** Medication use by patients in the MDD group.

**Medication**	**MDD (*n* = 55)**
**Antidepressants**, ***n*** **(%)**	55 (100.00%)
SSRI *(sertraline, escitalopram oxalate, fluvoxamine)*	45 (81.8%)
SNRI *(duloxetine)*	2 (3.6%)
NDRI *(bupropion)*	5 (9.1%)
Others *(vortioxetine, agomelatine)*	3 (5.5%)
**Antipsychotics**, ***n*** **(%)**	32 (58.2%)
*Aripiprazole*	11 (20.0%)
*Lurasidone*	7 (12.7%)
*Quetiapine*	8 (14.5%)
Others *(olanzapine, paliperidone, peropirox)*	6 (10.9%)
**Mood stabilizers**, ***n*** **(%)**	18 (32.7%)
*Lithium*	7 (12.7%)
*Sodium valproate*	6 (10.9%)
*Lamotrigine*	5 (9.1%)

SSRI, selective serotonin reuptake inhibitor; SNRI, serotonin-norepinephrine reuptake inhibitor; NDRI, norepinephrine-dopamine reuptake inhibitor.

### Diagnostic efficacy

**Figure 1:**
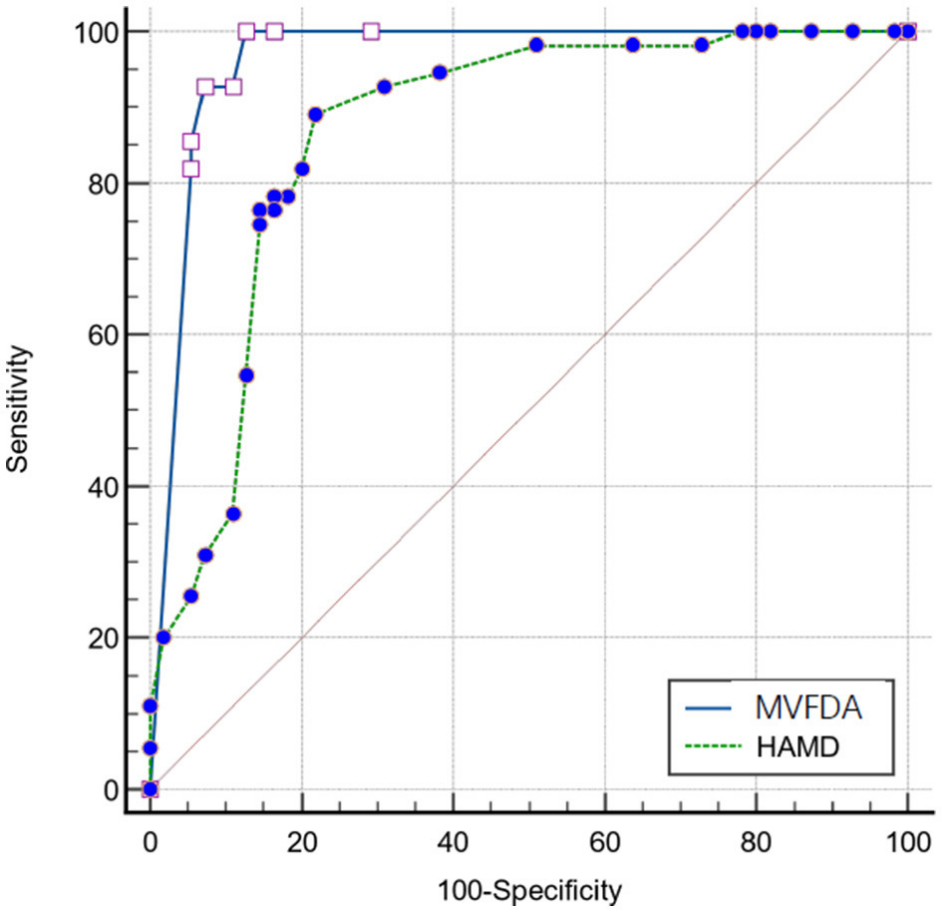
Receiver operator characteristic curve of the MVFDA and HAMFDA.

The kappa analysis result was 0.964, indicating that the HAMD scale findings were reliable and had a good agreement. As shown in Table 3, in the MDD group, the MVFDA system's correct/incorrect diagnosis was 51/4, and the HAMD scale's correct/incorrect diagnosis was 43/12; in the control group, the MVFDA system's correct/incorrect diagnosis was 50/5, and the HAMD scale's correct/incorrect diagnosis was 47/8. The evaluation indices are shown in Table 4. The sensitivity (92.73 vs. 76.36%, *P* = 0.04) and the specificity (90.91 vs. 85.45%, *P* > 0.05) of MVFDA system are significantly and slightly higher than those of the HAMD scale. However, the difference is not statistically significant. The Youden index, likelihood ratios, predictive value, and diagnostic accuracy of the MVFDA system are also higher than the corresponding items of the HAMD, as shown in Table 3. In addition, the ROC curves of MVFDA and HAMD are shown in Figure 1. The AUC of the MVFDA (0.962) was greater than that of the HAMD (0.962), and the difference between them was statistically significant (*p* = 0.012), indicating that the diagnostic efficacy of the MVFDA system is significantly higher than that of the HAMD.

**Table 3:** MVFDA system and HAMD results for all subjects.

	**MVFDA system**		**HAMD**	
	**Positive (** ***n*** **)**	**Negative (** ***n*** **)**	**Positive (** ***n*** **)**	**Negative (** ***n*** **)**
MDD group (*n* = 55)	51	4	43	12
HC group (*n* = 55)	5	50	8	47
Total (*n* = 110)	56	54	51	59

**Table 4:** Calculation results of the diagnostic efficacy of MVFDA and HAMD.

	**Sensitivity (%)**		**Specificity (%)**		**Youden index**	**PLR**		**NLR**		**Diagnostic odds ratio**	**PPV (%)**		**NPV (%)**		**Diagnostic accuracy (%)**
	**Point value**	**95%CI**	**Point value**	**95%CI**		**Point value**	**95%CI**	**Point value**	**95%CI**		**Point value**	**95%CI**	**Point value**	**95%CI**	
MVFDA	92.73	81.57–97.64	90.91	79.30–96.60	0.84	10.2	4.40–23.60	0.08	0.03–0.20	127.5	91.07	79.63–96.67	92.59	81.26–97.60	91.82
HAMD	76.36	70.70–91.80	85.45	77.07–95.49	0.62	5.25	3.57–16.46	0.28	0.10–0.34	18.98	80.77	75.97–95.22	81.03	72.07–92.23	80.91

NLR, negative likelihood ratio; NPV, negative predictive value; PLR, positive likelihood ratio; PPV, positive predictive value.

## Discussion

A novel voiceprint retrieval algorithm is proposed for the diagnosis of depression in children and adolescents. We found that the developed MVFDA system in this paper has exceptional diagnostic utility. Compared to the voiceprint system, the HAMD scale has lower sensitivity, likelihood ratios, predictive accuracy, and other indicators. This result is due to the limitations of the HAMD scale in its clinical application to children and adolescents with depressive disorders. The HAMD has the following drawbacks: (1) The evaluation takes a long time. The HAMD evaluation process takes at least 15–20min, it can be challenging for children and adolescents with depression to maintain steady attention during the interviews, which affects the quality of the evaluation (28). (2) The Hamilton Depression Scale raters are highly professional, necessitating professional, consistent training to ensure the quality of evaluation (29, 30). Compared with the typical depressive symptoms of adults, the clinical features of children and adolescents with depression are relatively complex. For example, adolescents with depressive disorders may be more likely to have physical complaints, psychomotor agitation, anxiety, and other manifestations, and they might have trouble responding to the item “depressed” (31). For inexperienced evaluators, it can often be difficult to accurately assess the patient's disease status, which leads to misestimation. (3) The level of the patient's cooperation must be high. As an interview measure, the HAMD scale requires effective communication between the evaluator and patient to gather medical history and assess clinical symptoms. Children and adolescents often struggle to describe their own disease condition accurately (10). Communication skills are required to get the patient to cooperate with the scale's assessment. If the patient is unwilling to cooperate with the assessment due to unfamiliarity or other reasons, the assessment of HAMD will not proceed smoothly.

The inadequacies of the HAMD may also be present in other scales; however, the ability to recognize depressive disorders through speech effectively overcomes these problems. First, the assessment phase of the MVFDA system is short, lasting less than 5 min, which means children “and adolescents” attention remains relatively steady. Second, the operation is simple. The auxiliary staff only needs to guide the subjects correctly to record their voices without any formal training. Third, without complex cooperation in question and answer with raters, the patients only complete the test by reading aloud the required paradigm. The data collected by the test is directly extracted from the patients. Analyzing the characteristics of the disease-related voice ensures the objectivity of the evaluation and prevents the subjectivity of the rater's evaluation.

**Figure 2:**
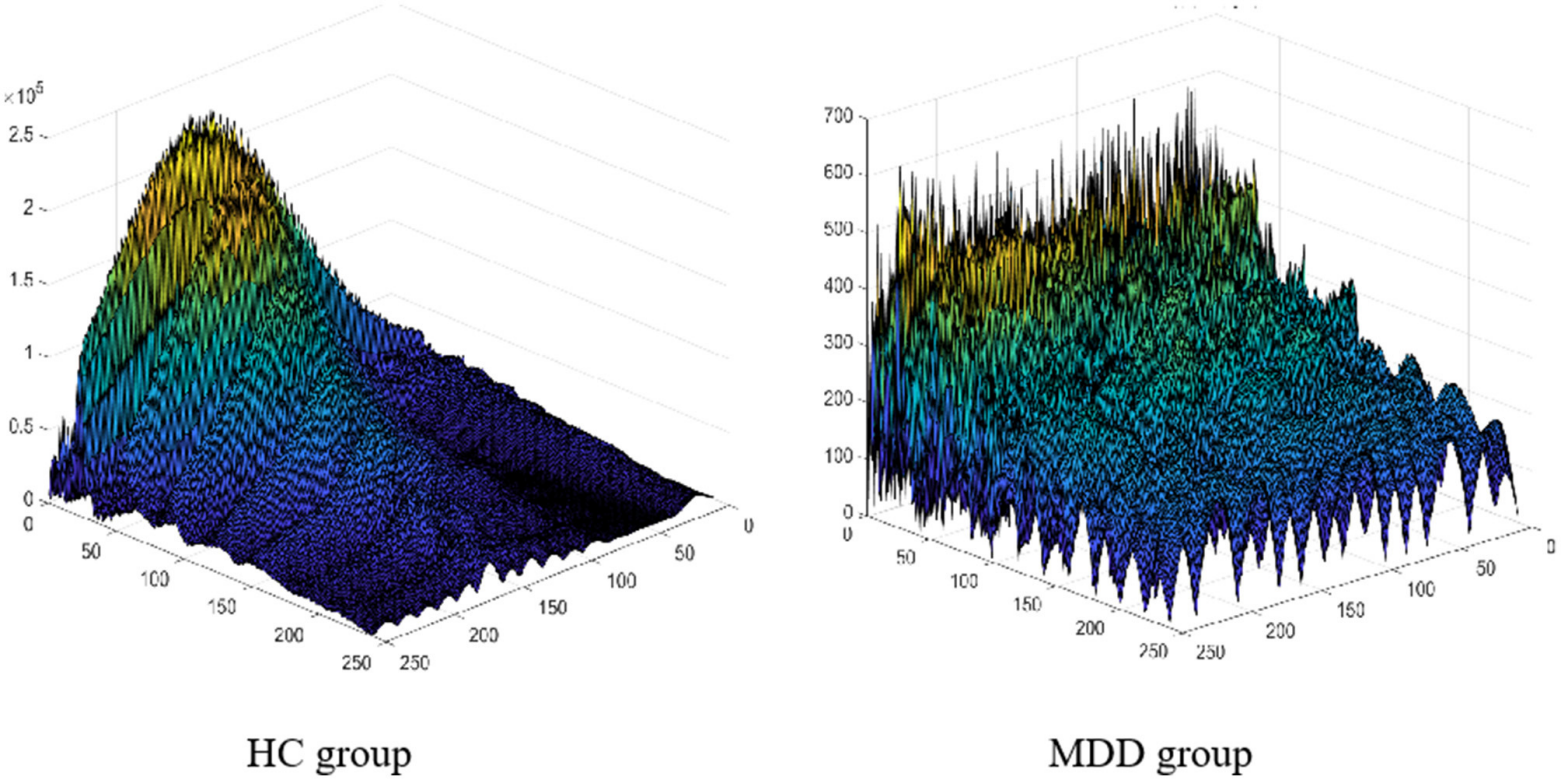
The feature visualization results of the HC and MDD groups.

In addition, the research findings that we developed based on the proposed algorithm could have a high degree of diagnostic accuracy for children and adolescents. In previous studies, the voiceprint system was mostly built based on data from adults with depression (32). However, due to the differences between adults, children, and adolescents, the voiceprints used in previous research were not compatible with our collected data. Furthermore, the number of voiceprint features used in state-of-the-art studies is small. Also, few attributes could deliver good results for specific groups, or even specific environments of audio acquisition, who bears poor robustness (33). In previous studies, a single voice was used as a sample, so multiple voices of the same person were used for both training and testing. Because the association between the voices of the same person is ignored, the accuracy of the system recognition is relatively high; however, in the actual application scenario, the accuracy is drastically decreased (34). To solve the aforementioned issues, we created an effective data set specifically for children and adolescents, based on resources acquired from the hospital. Additionally, in terms of feature extraction, LLD and its statistical features related to energy, spectrum, and rhythm were used to obtain a more comprehensive feature set, breaking the defect of a single feature promoted by existing methods, which leads to the difficulty in extracting common features of patients with depression. In addition to the time and frequency domains, our algorithm is extended to the wavelet domain to maximize the difference between the two groups of people, as shown by the feature visualizations in Figure 2. To achieve effective feature screening, the proposed algorithm applies KS test combined with maximum information coefficient (MIC), where KS test is used to screen features with large differences between classes, and MIC is used to remove features with high correlation. Finally, based on sufficient samples, and taking into account the correlation among the voices of the same person, the proposed algorithm treats a person as a sample when training the model, and the voice of the same person can only be applied for training or testing to achieve higher accuracy, which is more consistent with the actual application scenarios. Improvements in ensuring the effective identification of children and adolescents with depressive disorder.

The MVFDA system is the first generation; hence, it has some shortcomings. First of all, this study used a single device to capture speech, which may not reflect the impact of different devices on recording quality. Second, the dimensionality of the features extracted by the algorithm is not sufficiently streamlined. In the future, another dataset might be established where speech is collected using multiple recording devices and in different environments to investigate the impact of recording devices and environments on the MVFDA. Besides, further consideration is given to screening and reducing redundant features in addition to ensuring high classification accuracy by maintaining the features with the greatest differences between classes.

This test verified the scientific validity and reliability of the algorithm, and the results showed that it is very suitable for clinical diagnosis and application promotion. The detection system can reduce the need for a depression examination facility. Under the premise of solving privacy issues and other issues, the complete voiceprint evaluation system could be used by families, schools, and primary medical units. Voices are collected through a simple paradigm. Data are collected and transferred to the cloud devices to obtain a report, which is then sent to the doctors for guidance. Its convenience and efficiency are used for large-scale early screening of MDD to reduce the medical burden and greatly improve the diagnostic efficiency of clinicians.

## Conclusions

The MVFDA system performed well in clinical diagnostic studies for identifying MDD in children and adolescents by capturing objective voiceprint features. The algorithm's scientific and dependable characteristics were verified. Simulations revealed that the sensitivity, the Youden Index, likelihood ratios, predictive value, and diagnostic accuracy of the MVFDA system were higher than those of the HAMD. The specificity of the MVFDA system was also slightly lower than that of the HAMD scale. Furthermore, considering the ROC and the AUC of the MVFDA system, the diagnostic efficacy of MVFDA is significantly higher than that of the HAMD. Compared with the scale assessment, the MVFDA system deserves to be further promoted in clinical practice for its advantages in terms of simple operation, objective evaluation, and high diagnostic efficiency.

## Data availability statement

The raw data supporting the conclusions of this article will be made available by the authors, without undue reservation.

## Ethics statement

The studies involving human participants were reviewed and approved by the Ethics Committee of Beijing Institute of Technology (Ethical Number: BIT-EC-H-2022120). Written informed consent to participate in this study was provided by the participants' legal guardian/next of kin.

## Author contributions

JLuo, ML, LF, ZL, YW, JLu, and FH are involved in the collection of the data. JLuo performed the data analysis and wrote the final manuscript. JLu made substantial revisions to the first draft of the manuscript. ML, LF, and ZL were involved throughout the data collection process. JLu was primarily involved in the design of the MVFDA system. FH made practical contributions to the conduct and development of the trial. All authors contributed to the article and approved the submitted version.

## References

[1] The global burden of disease: 2004 update. Geneva: World Health Organization [EB/OL].

[2] Avenevoli S Swendsen J He J-P Burstein M Merikangas KR. Major depression in the national comorbidity survey-adolescent supplement: prevalence, correlates, and treatment. J Am Acad Child Adolesc Psychiatry. (2015) 54:37–44.e2. 10.1016/j.jaac.2014.10.010 25524788 PMC4408277

[3] Dwyer JB Stringaris A Brent DA Bloch MH. Annual research review: defining and treating pediatric treatment-resistant depression. J Child Psychol Psychiatry. (2020) 61:312–32. 10.1111/jcpp.13202 32020643 PMC8314167

[4] Asarnow JR Baraff LJ Berk M Grob C Devich-Navarro M Suddath R. Pediatric emergency department suicidal patients: two-site evaluation of suicide ideators, single attempters, and repeat attempters. J Am Acad Child Adolesc Psychiatry. (2008) 47:958–66. 10.1097/CHI.0b013e3181799ee8 18596552

[5] Weissman MM Wolk S Goldstein RB Moreau D Adams P Greenwald S. Depressed adolescents grown up. JAMA. (1999) 281:1707–13. 10.1001/jama.281.18.1707 10328070

[6] Fergusson DM Woodward LJ. Mental health, educational, and social role outcomes of adolescents with depression. Arch Gen Psychiatry. (2002) 59:225–31. 10.1001/archpsyc.59.3.225 11879160

[7] Hamilton M. The Hamilton Rating Scale for Depression. In Sartorius N, Ban TA, editor. Assessment of Depression, Berlin, Heidelberg: Springer Berlin Heidelberg. 1986:143-152. 10.1007/978-3-642-70486-4_14

[8] Faries D Herrera J Rayamajhi J DeBrota D Demitrack M Potter WZ. The responsiveness of the Hamilton depression rating scale. J Psychiatr Res. (2000) 34:3–10. 10.1016/S0022-3956(99)00037-0 10696827

[9] Bagby RM Ryder AG Schuller DR Marshall MB. The Hamilton depression rating scale: has the gold standard become a lead weight? Am J Psychiatry. (2004) 161:2163–77. 10.1176/appi.ajp.161.12.2163 15569884

[10] Rice F Riglin L Lomax T Souter E Potter R Smith DJ. Adolescent and adult differences in major depression symptom profiles. J Affect Disord. (2019) 243:175–81. 10.1016/j.jad.2018.09.015 30243197

[11] Miller L Campo JV. Depression in adolescents. N Engl J Med. (2021) 385:445–9. 10.1056/NEJMra2033475 34320289

[12] Malhi GS Mann JJ. Depression. Lancet. (2018) 392:2299–312. 10.1016/S0140-6736(18)31948-2 30396512

[13] Pampouchidou A Simantiraki O Vazakopoulou C-M Chatzaki C Pediaditis M Maridaki A. Facial geometry and speech analysis for depression detection. Annu Int Conf IEEE Eng Med Biol Soc. (2017) 2017:1433–6. 10.1109/EMBC.2017.8037103 29060147

[14] Mundt JC Vogel AP Feltner DE Lenderking WR. Vocal acoustic biomarkers of depression severity and treatment response. Biol Psychiatry. (2012) 72:580–7. 10.1016/j.biopsych.2012.03.015 22541039 PMC3409931

[15] Wu P Wang R Lin H Zhang F Tu J Sun M. Automatic depression recognition by intelligent speech signal processing: a systematic survey. Caai Transactions on Intelligence Technology. (2022). 10.1049/cit2.12113

[16] Graham S Depp C Lee EE Nebeker C Tu X Kim H-C. Artificial intelligence for mental health and mental illnesses: an overview. Curr Psychiatry Rep. (2019) 21:116. 10.1007/s11920-019-1094-0 31701320 PMC7274446

[17] Szabadi E Bradshaw CM Besson JaO. Speech in depressive states. BJPsych. (1977) 131:109–10. 10.1192/S0007125000013507 884406

[18] Greden JF Carroll BJ. Decrease in speech pause times with treatment of endogenous depression. Biol Psychiatry. (1980) 15:575–87. 7397288

[19] Hollien H. Vocal indicators of psychological stress. Ann NY Acad Sci. (1980) 347:47–72. 10.1111/j.1749-6632.1980.tb21255.x 6930921

[20] Ooi KEB Lech M Allen NB. Multichannel weighted speech classification system for prediction of major depression in adolescents. IEEE Transactions on Biomedical Engineering. (2013) 60:497–506. 10.1109/TBME.2012.2228646 23192475

[21] Cummins N Epps J Breakspear M Goecke R. An investigation of depressed speech detection: features and normalization[C]. 12th Annual Conference of the International-Speech-Communication-Association. (2011) 2011:2997–3000. 10.21437/Interspeech.2011-750

[22] Mendiratta A Scibelli F Esposito AM Capuano V Likforman-Sulem L Maldonato MN. Automatic Detection of Depressive States from Speech. In: Multidisciplinary Approaches to Neural Computing. Cham: Springer International Publishing (2018). p. 301–14. 10.1007/978-3-319-56904-8_29

[23] Hussenbocus AY Lech M Allen NB. Statistical differences in speech acoustics of major depressed and non-depressed adolescents. In: 9th International Conference on Signal Processing and Communication Systems (ICSPCS). (2015). 10.1109/ICSPCS.2015.7391781

[24] Aben I Verhey F Lousberg R Lodder J Honig A. Validity of the beck depression inventory, hospital anxiety and depression scale, SCL-90, and Hamilton depression rating scale as screening instruments for depression in stroke patients. Psychosomatics. (2002) 43:386–93. 10.1176/appi.psy.43.5.386 12297607

[25] Pan S Liu Z-W Shi S Ma X Song W-Q Guan G-C. Hamilton rating scale for depression-24 (HAM-D(24)) as a novel predictor for diabetic microvascular complications in type 2 diabetes mellitus patients. Psychiatry Res. (2017) 258:177–83. 10.1016/j.psychres.2017.07.050 28774662

[26] Zhu G Yin Y Xiao C-L Mao R-J Shi B-H Jie Y. Serum DHEAS levels are associated with the development of depression. Psychiatry Res. (2015) 229:447–53. 10.1016/j.psychres.2015.05.093 26205628

[27] Sun XY Li YX Yu CQ Li LM. Reliability and validity of depression scales of Chinese version: a systematic review. Zhonghua Liu Xing Bing Xue Za Zhi. (2017) 38:110−16. 10.3760/cma.j.issn.0254-6450.2017.01.021 28100388

[28] Müller MJ Dragicevic A. Standardized rater training for the Hamilton Depression Rating Scale (HAMD-17) in psychiatric novices. J Affect Disord. (2003) 77:65–9. 10.1016/S0165-0327(02)00097-6 14550936

[29] Cicchetti DV Prusoff BA. Reliability of depression and associated clinical symptoms. Arch Gen Psychiatry. (1983) 40:987–90. 10.1001/archpsyc.1983.01790080069009 6351786

[30] Demitrack MA Faries D Herrera JM DeBrota D Potter WZ. The problem of measurement error in multisite clinical trials. Psychopharmacol Bull. (1998) 34:19–24. 9564194

[31] Loades ME Clair MCS Orchard F Goodyer I Reynolds S I CONSORTIUM. Depression symptom clusters in adolescents: a latent class analysis in a clinical sample. Psychother Res. (2022) 32:860–73. 10.1080/10503307.2022.2030498 35109777

[32] Yalamanchili B Kota NS Abbaraju MS Nadella VSS Alluri SV. Real-time acoustic based depression detection using machine learning techniques[C]// (2020). International Conference on Emerging Trends in Information Technology and Engineering (ic-ETITE). (2020). 10.1109/ic-ETITE47903.2020.39435432567

[33] Low L-SA Maddage NC Lech M Sheeber LB Allen NB. Detection of clinical depression in adolescents' speech during family interactions. IEEE Trans Biomed Eng. (2010) 58:574–86. 10.1109/TBME.2010.2091640 21075715 PMC3652557

[34] Zhao Y Liang Z Du J Zhang L Liu C Zhao L. Multi-Head attention-based long short-term memory for depression detection from speech. Front Neurorobot. (2021) 2021:111. 10.3389/fnbot.2021.684037 34512301 PMC8426553

